# Re‐reading of OraQuick HIV‐1/2 rapid antibody test results: quality assurance implications for HIV self‐testing programmes

**DOI:** 10.1002/jia2.25234

**Published:** 2019-03-25

**Authors:** Victoria Watson, Russell J Dacombe, Christopher Williams, Thomas Edwards, Emily R Adams, Cheryl C Johnson, Miriam N Mutseta, Elizabeth L Corbett, Frances M Cowan, Helen Ayles, Karin Hatzold, Peter MacPherson, Miriam Taegtmeyer

**Affiliations:** ^1^ Department of International Public Health Liverpool School of Tropical Medicine Liverpool United Kingdom; ^2^ Research Centre for Drugs and Diagnostics Liverpool School of Tropical Medicine Liverpool United Kingdom; ^3^ HIV Department World Health Organisation Geneva Switzerland; ^4^ Population Services International Harare Zimbabwe; ^5^ Department of Clinical Research London School of Hygiene & Tropical Medicine London United Kingdom; ^6^ Malawi Liverpool Welcome Trust Clinical Research Programme Blantyre Malawi; ^7^ Centre for Sexual Health HIV and AIDS Research Harare Zimbabwe; ^8^ Zambart Lusaka Zambia; ^9^ Department of Clinical Sciences Liverpool School of Tropical Medicine Liverpool United Kingdom

**Keywords:** HIV self‐testing, Quality assurance, Delayed re‐reading, Visual stability, False reactive, Misdiagnosis, HIV testing

## Abstract

**Introduction:**

Scale‐up of HIV self‐testing (HIVST) will play a key role in meeting the United Nation's 90‐90‐90 targets. Delayed re‐reading of used HIVST devices has been used by early implementation studies to validate the performance of self‐test kits and to estimate HIV positivity among self‐testers. We investigated the stability of results on used devices under controlled conditions to assess its potential as a quality assurance approach for HIVST scale‐up.

**Methods:**

444 OraQuick^®^ HIV‐1/2 rapid antibody tests were conducted using commercial plasma from two HIV‐positive donors and HIV‐negative plasma (high‐reactive n = 148, weak‐reactive n = 148 and non‐reactive n = 148) and incubated them for six months under four conditions (combinations of high and low temperatures and humidity). Devices were re‐read daily for one week, weekly for one subsequent month and then once a month by independent readers unaware of the previous results. We used multistage transition models to investigate rates of change in device results, and between storage conditions.

**Results and discussion:**

There was a high incidence of device instability. Forty‐three (29%) of 148 initially non‐reactive results became false weak‐reactive results. These changes were observed across all incubation conditions, the earliest on Day 4 (n = 9 kits). No initially HIV‐reactive results changed to a non‐reactive result. There were no significant associations between storage conditions and hazard of results transition. We observed substantial statistical agreement between independent re‐readers over time (agreement range: 0.74 to 0.96).

**Conclusions:**

Delayed re‐reading of used OraQuick^®^ HIV‐1/2 rapid antibody tests is not currently a valid methodological approach to quality assurance and monitoring as we observed a high incidence (29%) of true non‐reactive tests changing to false weak‐reactive and therefore its use may overestimate true HIV positivity.

## 1 Introduction

HIV self‐testing (HIVST) is being scaled‐up using a variety of distribution models throughout Africa, the Americas, Asia and Europe 1, 2, 3, 4. No clear monitoring and evaluation or external quality assurance (EQA) systems exist for HIVST devices and this raises concern for national reference laboratories, regulators and policymakers 5, 6, 7.

While previous studies report acceptable sensitivity and specificity when HIVST is conducted by intended users 8, 9, it is unclear whether this will be maintained once HIVST programmes are implemented at scale. Observation and in‐depth interviews reveal that without a demonstration, operator errors are common in both conducting and interpreting self‐tests 10, 11. Scale‐up will have to be accompanied by a robust quality assurance system.

A reactive HIVST indicates that HIV antibodies are present in the oral or fingerstick/blood sample of the user. Further testing to confirm a positive diagnosis following linkage to care acts as an active system for detecting false‐reactive results and ensures individuals are not incorrectly started on antiretroviral therapy (ART). In most contexts, however, the prevalence of false‐reactive results prior to ART clinic enrolment (whether or not individuals came from HIVST) is not tracked and rates of linkage remain highly variable and can be very low without active support 12, 13, 14. Self‐testers with non‐reactive results, unless linking to voluntary male medical circumcision or pre‐exposure prophylaxis services, would not typically seek or receive further testing and confirmation, meaning a false non‐reactive result would not be detected.

Methods that detect incorrect results and misinterpretation are required for individual care as well as for quality assurance. One approach, which has been utilized in early HIVST implementation studies, is for self‐testers to return used devices for delayed re‐reading by trained staff in parallel with self‐reported interpretation of results 15, 16. However, delays between device use and re‐reading, and environmental storage conditions during this period could impair the validity of this method. We therefore set out to investigate the stability of OraQuick^®^ HIV‐1/2 rapid antibody test (OraQuick HIV) results with delayed re‐reading stored under controlled incubation conditions for prolonged periods. We selected the OraQuick^®^ HIV‐1/2 rapid antibody test kit, which is the same product (in different packaging) as the OraQuick^®^ HIV Self‐Test which is prequalified by the World Health Organization (WHO) 17.

## 2 Methods

### 2.1 Materials and equipment

Two different batches (HIVCO‐4308 and HIVCO‐4309) of OraQuick^®^ HIV‐1/2 rapid antibody test kits (assembled in Thailand for OraSure Technologies, Inc. Bethlehem, PA, USA) were obtained from the manufacturer. Human HIV seroconversion panel plasma samples from two donors (Donor No. 73695 panel number 12007‐08 and 09, 18 and 75018 panel number 9077‐24 and 25 19) were purchased from ZeptoMetrix Corporation (Buffalo, NY, USA). Human plasma negative for HIV, hepatitis B, C, E and syphilis was purchased from the National Blood Service (Liverpool, UK).

### 2.2 Sample preparation

The OraQuick^®^ HIV‐1/2 Rapid Antibody Test is WHO prequalified for use with oral fluid, whole blood, serum or plasma. The matrix of the sample (i.e. plasma rather than an oral crevicular fluid sample) was not a crucial factor in this investigation, as we were not investigating specificity or sensitivity. What was important was the basis of the immuno‐chromatographic stability of the test. The use of HIV antibody‐positive and ‐negative plasma allowed us to investigate this.

Four panel samples from two donors were combined to produce an HIV‐reactive “mini‐pool” of stock serum. This was checked to ensure the correct result and intensity of test line on the OraQuick HIV device. From this stock, an HIV‐reactive sample was prepared with the addition of HIV‐negative plasma (1:8 dilution factor). An HIV weak‐reactive sample was prepared with a 1:16 dilution factor.

### 2.3 Sample size calculation

To estimate sample size, we assumed that 0.2% of all used tests would change over six months. To estimate accuracy of rate of change within ± 1% with 95% confidence, 77 tests were required to be read for each condition, with a total of four conditions, giving a minimum sample of 308 kits. With available resources, were able to include more samples (444 total).

### 2.4 Conducting the tests

The study was conducted in the laboratory under controlled conditions rather than using actual patient‐used HIVST devices. This eliminated the risk that the test had not been performed correctly which could have influenced the study results.

A total of 444 OraQuick HIV tests were conducted in the laboratory following the manufacturer's instructions for use (IFU). Five microlitres of the prepared samples (HIV reactive n = 148, HIV weak‐reactive n = 148 or HIV non‐reactive n = 148) was delivered into the developer solution before mixing gently. The test device was labelled with an identification number on the back and inserted “pad end” into the developer solution. Devices were read within the 20‐ to 40‐minute reading window (measured using a digital timer) by three different laboratorians, trained in the reading of the devices and blinded to each other's interpretation.

### 2.5 Read definitions/interpretation

On the test device there is a window next to which there is a letter “T” for test line and a “C” for control line. As per the IFU, a non‐reactive result was recorded when only a single quality control line was visible adjacent to the letter “C” on the test device. A weak‐reactive was recorded when there were two visible lines on the test device, the first adjacent to the letter “C” (control) and the second adjacent to the letter “T” (test) but the test line was not as intense as the control line. A reactive test was recorded when both “C” and “T” lines were visible and the “T” line was at least as intense as the “C” line. An invalid result was defined as no line present adjacent to the letter “C.”

### 2.6 Incubation conditions

**Figure 1:**
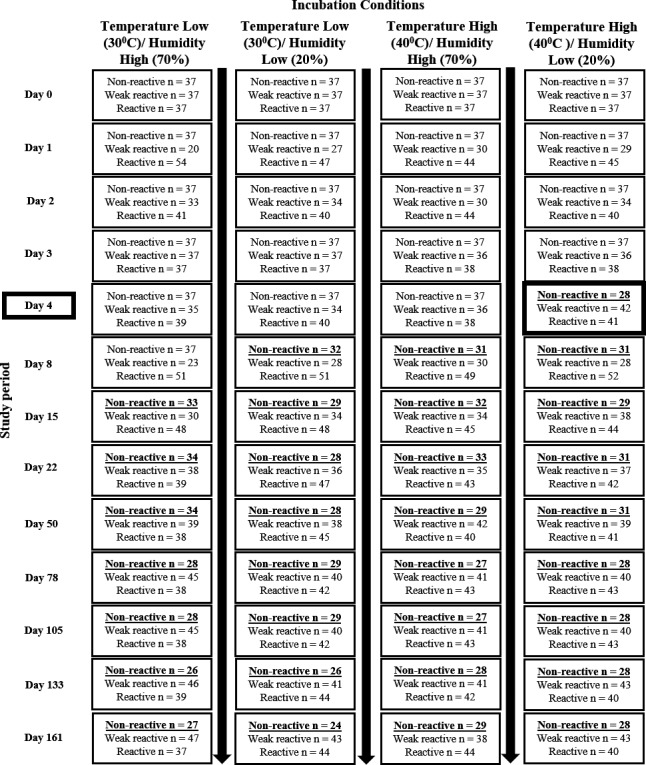
Flow diagram of sample allocation and re‐read results over time The flow chart shows the allocation of non‐reactive, weak reactive and reactive test devices to the four different incubation conditions on Day 0 and the re‐read results for Day 0 to Day 161. Changes in non‐weak reactives are underlined and highlighted in bold. The first changes observed “non‐reactive” transitioning to “weak reactive” was on Day 4 in the incubation condition of high temperature and low humidity.

Following initial reads, devices were placed in one of four laboratory benchtop incubators (Benchmark Scientific), each set to a different incubation condition: (a) control temperature (30°C) with high humidity (70%); (b) control temperature (30°C) with low humidity (20%); (c) high temperature (40°C) with high humidity (70%); (d) or high temperature (40°C) with low humidity (20%). Each condition had 37 HIV non‐reactive, 37 HIV weak‐reactive and 37 HIV reactive devices allocated to it (Figure 1).

### 2.7 Re‐reading intervals

Devices were re‐read by either two or three blinded and independent readers daily for one week, weekly for one subsequent month and then once a month for the following five months, giving a total of 13 reads over the 6‐month study period (November 2016 through to April 2017). Each of the laboratorians interpreted the test face up, recorded the test result (check box non‐reactive, weak‐reactive or reactive) on the data log sheet and then turned the test over to record the test identification number along with any additional comments. Data were input onto a blinded (of previous re‐read result) electronic log. Data were unblinded and analysed after 6 months.

### 2.8 Data analysis

Two laboratorians had to be in agreement for a “final” test result interpretation. We compared agreement between readers at each time point using the kappa statistic with bootstrapped 95% confidence intervals for three readers and Scott's pi for two readers. To estimate the hazard of transition between device states (non‐reactive, weak‐reactive, and reactive) over time, and the effects of incubation storage conditions, we fitted a multistage transition model using a hidden Markov process. Model fit was evaluated by visually comparing the fitted hazard function within each condition over time with observed transition events. In the final model, terms for piecewise intensities were fitted at Day 1 to 2, Day 2 to 3; Day 3 to 4; Day 8 to 15; and Day 15 to 181 to account for the high intensity of transition. Analysis was done using R version 3.3.2 (R Foundation for Statistical Computing, Vienna).

## 3 Results and discussion

Devices were first read following the manufacturer IFU, after 20 minutes and within 40 minutes of conducting the test (Day 0) for control purposes. On Day 0, all reactive devices gave the expected dilution results (reactive or weak‐reactive) and a following masked re‐read showed all three independent readers in agreement (100%). Statistical agreement between independent readers over the six‐month period ranged from 0.70 (95% confidence interval (CI): 0.66 to 0.74) to 0.96 (95% CI: 0.94 to 0.98) 20.

**Figure 2:**
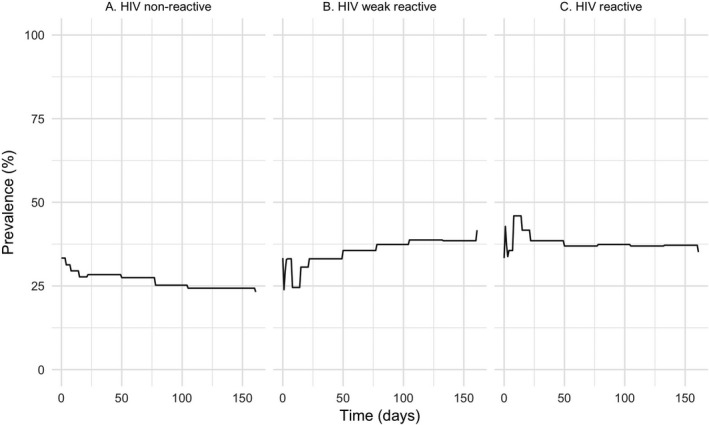
Observed transitions between HIV re‐read results over the study period (A) Decrease in true HIV non‐reactive inoculated test devices as they transit to “false” HIV weak reactive. (B) Increase in the number of test devices re‐read as HIV weak reactive. (C) Increase in the number of test devices re‐read as reactive which then transition back to weak reactive over time.

There was a high incidence of OraQuick HIV result transition between states over time (Figure 2). A total of 43 of the 148 true non‐reactive devices (29%) changed to a false weak‐reactive result with the earliest change observed on Day 4 (n = 9 kits) incubated at high temperature and low humidity (Figure 1). Transition between states over time was also observed, with tests changing from true non‐reactive to false weak‐reactive and then back to true non‐reactive (77 instances out of a total of 1776) and weak‐reactive results changing to strong reactive and then back to weak‐reactive (135 instances). The majority of these true reactive transitions occurred early (from Day 1) with the greatest intensity of transition occurring up to Day 15. Transitions continued to occur throughout the six‐month follow‐up period. No devices with an initial reactive result changed to a weak‐reactive or non‐reactive result over the six‐month period. The test control line showed 100% stability throughout the study.

Changes occurred across all controlled incubation conditions with the earliest transition from a true non‐reactive to a false weak‐reactive occurring under high temperature and low humidity conditions on Day 4. However, in our final model, there was no significant association between the incubation condition under which devices were stored and the hazard of transition between stages (Table 1).

**Table 1:** Hazard of transition between HIV test read stage over six months

Incubation condition and transition stage (From → To)	Hazard ratio for transition intensity (vs. cool/dry incubation condition)	95% confidence interval
Cool/humid		
HIV non‐reactive → HIV weak‐reactive	0.88	0.47 to 1.64
HIV weak‐reactive → HIV non‐reactive	0.95	0.33 to 2.71
HIV weak‐reactive → HIV reactive	1.27	0.80 to 2.03
HIV reactive → HIV weak‐reactive	1.41	0.87 to 2.28
Warm/humid		
HIV non‐reactive → HIV weak‐reactive	0.81	0.43 to 1.56
HIV weak‐reactive → HIV non‐reactive	1.27	0.47 to 3.42
HIV weak‐reactive → HIV reactive	0.87	0.53 to 1.44
HIV reactive → HIV weak‐reactive	0.94	0.56 to 1.59
Warm/Dry		
HIV non‐reactive → HIV weak‐reactive	1.06	0.57 to 1.95
HIV weak‐reactive → HIV non‐reactive	1.24	0.46 to 3.35
HIV weak‐reactive → HIV reactive	0.94	0.58 to 1.52
HIV reactive → HIV weak‐reactive	1.15	0.69 to 1.89

Estimated by fitting multistage transition model for each test read condition with hidden Markov process, and with terms for incubation condition and piecewise transition intensities between Day 1 to 2, Day 2 to 3, Day 3 to 4, Day 8 to 15 and Day 15 to 181.

Our key finding shows the OraQuick HIV device can have a result change from a true non‐reactive to a false weak‐reactive result when reading is extended beyond the manufacturer reading time window. The reasons underlying our finding are not clear and we did not find any association with different temperature and humidity conditions. Explanations for the appearance of the false weak‐reactive lines may be due to nonspecific antibody binding at the HIV antigen test site on the devices nitrocellulose test strip 21 or nonspecific binding of protein‐A gold conjugate which the test uses as the colorimetric indicator or perhaps a lateral back flow or “settling effect” over time and further investigation into these hypotheses is required.

The observed change in result raises concerns over the use of delayed re‐reading of devices for monitoring HIVST interpretation, as well as for programmatic monitoring, evaluation and EQA. Research studies utilizing delayed re‐reading of returned OraQuick^®^ HIV Self‐Test for establishing positivity may overestimate the true HIV positivity amongst a self‐testing population.

A previous study conducted in Malawi examined the pre‐use stability of OraQuick^®^ HIV test kits 16. 371 optimally stored and 375 pre‐incubated used devices were re‐read over a 12‐month period. A 0.2% change from an initial reactive result to a later non‐reactive was observed (one in the pre‐incubated and one in the optimally stored group). These results suggested that HIVST device results remained stable over time. However, the focus of this study was its effect on pre‐use storage conditions. Post‐use storage conditions were not rigorously monitored and so cannot be reliable compared with the results from our study.

During this controlled study, our trained laboratorians could correctly distinguish false weak‐reactive test lines from true weak‐reactive test lines as they have a greyish appearance compared with the pinker true‐reactive. Implementation of this more nuanced approach may however prove challenging in programmatic settings where previous reports show that providers struggle to identify and interpret weak reactives 22 and other factors, such as interferents, and tests used among people with HIV using ART can cause weak reactives 23.

In addition to a false weak‐reactive line causing uncertainty to an EQA model, when testing a population, it is likely that more “true negative” samples will change to “false weak reactive” and delayed re‐reading by self‐testers themselves could lead to individual misinterpretation and misunderstandings. Our study showed that the OraQuick HIV device was stable up to four days after the sample was applied, suggesting that the risk of this is low but nevertheless self‐testers need clear messages about the read window and the importance of reading the device according to manufacturer instructions.

A limitation of this study was that on some re‐read days only two individual re‐reads were conducted (23%) and therefore a third “tie breaker” re‐read was not available. The very nature of self‐testing (conducting the test privately at home) means conventional facility/laboratory‐based QA systems of test devices are eluded, and an alternative approach is required. Digital photography and immediate re‐reading are two other options that are being further explored for QA during HIVST scale‐up, but these also have their limitations. National reference laboratories should play an integral role in external quality control measures by conducting batch testing at the actual sites of distribution of HIVST to ensure that the integrity of the devices is not compromised during transport and storage.

## 4 Conclusions

The use of re‐reading used OraQuick HIVST devices as an approach to quality assurance and monitoring test results is not advised. The instability observed in true non‐reactive tests changing to false weak reactive test results in our study demonstrates that re‐reading is not a reliable method to assess user interpretation of the OraQuick HIVST and measurement of HIV positivity rates among self‐testers.

## Competing interests

PM is funded by the Wellcome Trust (206575/Z/17/Z). ELC is funded by the Wellcome Trust (WT200901/Z/16/Z). The remaining authors have no conflicts of interest to disclose.

## Authors’ contributions

VW, RD and MT formulated and designed the experiments. VW, RD, CW, TE and EA performed the experiments. VW, PM and RD analysed the data. VW, RD, CW, TE, EA, CJ, MM, EC, FC, HA, KH, PM and MT wrote the concise communication.
